# Gridded daily 2-m air temperature dataset for Ethiopia derived by debiasing and downscaling ERA5-Land for the period 1981–2010

**DOI:** 10.1016/j.dib.2022.108844

**Published:** 2022-12-20

**Authors:** Mosisa Tujuba Wakjira, Nadav Peleg, Paolo Burlando, Peter Molnar

**Affiliations:** a Institute of Environmental Engineering, ETH Zurich, Stefano-Franscini-Platz 5, CH-8093 Zurich, Switzerland; b Institute of Earth Surface Dynamics, University of Lausanne, CH-1015 Lausanne, Switzerland

**Keywords:** Climate data, Linear regression, Inverse distance weighting, Spatial interpolation, Bias correction, Quantile mapping

## Abstract

A gridded maximum and minimum (Tx and Tn) daily temperature dataset derived by spatial downscaling and bias correction of the ERA5-Land (ERA5L) for the period 1981–2010 is presented. Observed daily Tx and Tn at 154 stations in Ethiopia covering record lengths of 5–30 years were used as a reference. The statistics that define the Gaussian distribution (mean and standard deviation) of Tx and Tn from the station observations were interpolated in space to create a monthly climatology and interannual statistics at 0.05° × 0.05° resolution using a hybrid interpolation approach that combines linear regression with topographic and location attributes, and non-Euclidean inverse distance weighting interpolation. The interpolated monthly and interannual statistics were then used to debias the ERA5L Tx and Tn using a quantile mapping approach. Leave-one-out cross-validation showed that the mean absolute errors in the corrected and downscaled daily temperatures are about 0.7 °C for Tx and 1.1 °C for Tn, reducing the statistical biases in the ERA5L Tx and Tn by 68% and 25% respectively. For monthly climatology, 40–64% of the biases were removed for Tx while for Tn the reductions range from 19% to 32%. The correction also improved commonly used indices for extremes like the probability of warm days, cold days, and warm nights, but overestimated the probability of cold nights. The presented open-access Tx and Tn dataset is a substantial improvement over existing gridded temperature datasets for Ethiopia, such as ERA5L and the Climate Hazards Infrared Temperature with Station (CHIRTS), and we suggest it is suitable for a wide range of environmental applications, e.g. in the fields of hydrology, agriculture, and ecology.

**Specifications table**

**Table:** 

Subject	Earth and planetary science
Specific subject area	Climate science, meteorology, hydrology, agriculture, forestry, and human and environmental-related subjects
Type of data	Raw Analyzed Derived
How the data were acquired	(1) Spatial downscaling of the raw ERA5-Land (ERA5L) maximum (Tx) and minimum (Tn) temperature from 0.1° × 0.1° to 0.05° × 0.05° using bilinear interpolation; (2) Spatial interpolation of the observed Tx and Tn statistics (mean and standard deviation) at 154 stations using an inverse generalized distance weighting technique; (3) Quantile mapping to adjust the statistical biases in the downscaled ERA5L Tx and Tn using the transfer function constructed from the observed temperature field; and (4) Evaluation of the dataset using the Leave-One-Out Cross-Validation method.
Data format	Figure Table Image NetCDF
Description of data collection	The daily temperature records for Ethiopia used as a reference for bias correction and spatial downscaling were obtained from 154 In-situ OBServed (IOBS) climate stations with record lengths ranging from 5 to 30 years between the period 1981–2010. The raw daily ERA5L Tx and Tn were retrieved from the hourly ERA5L 2-m air temperature.
Data source location	Raw ERA5L Tx and Tn dataset source: • Institution: European Center for Medium-Range Weather Forecast (ECMWF) • Data center: Copernicus Climate Change Service (C3S) Climate Data Store (CDS) • Website: https://cds.climate.copernicus.eu/cdsapp#!/dataset/reanalysis-era5-land?tab=form Observed Tx and Tn data source for stations in Ethiopia: • Institution: National Meteorological Service Agency of Ethiopia • City: Addis Ababa • Country: Ethiopia Observed Tx and Tn data source for stations in the neighboring countries: • Institution: National Oceanic and Atmospheric Administration (NOAA) • Dataset: Global Historical Climatology Network (GHCN) daily • Website: https://www.ncei.noaa.gov/products/land-based-station/global-historical-climatology-network-daily
Data accessibility	Dataset file name: BCE5.zip Repository name: ETH Zurich Research Collection Data identification number: 10.3929/ethz-b-000546574 Direct permanent URL to data: https://doi.org/10.3929/ethz-b-000546574.

## Value of the Data

• The Bias-Corrected ERA5-Land (hereafter BCE5) temperature data is downscaled to a spatial resolution that matches common gridded precipitation products (such as the CHIRPS rainfall). Thus, it is highly convenient for use in combination with these datasets in a wide range of applications at national, regional, catchment and local scales.
• Compared to the raw ERA5L, BCE5 has an improved accuracy that is assimilated from the local temperature gradient at 154 ground observation stations, because the temperature statistics from the stations provide an unprecedented local temperature space-time variation for Ethiopia not incorporated in other gridded temperature datasets.
• The mean absolute errors in the BCE5 daily climatology are about 0.7 °C for Tx and 1.1 °C for Tn, reducing the statistical biases in the ERA5L Tx and Tn by 68% and 25% respectively. For monthly climatology, 40–64% of the biases were removed for Tx and while for Tn the reductions range from 19% to 32%.
• The BCE5 dataset is useful for researchers, practitioners, planners and decision-makers in various disciplines, for instance, agriculture, hydrology, and ecology, where weather and climate data are crucial.
• In particular, it can be used in climate impact assessment, heatwave and drought assessment, catchment hydrological modeling, crop growth modeling and monitoring, agricultural water management, water resources planning, ecological system monitoring, and understanding the complex water-energy-food-environment nexus in Ethiopia.

## 1 Data Description

### 1.1 The bias-corrected dataset

The bias-corrected temperature dataset presented in this paper has a spatial resolution of 0.05° × 0.05°. It is available at daily time steps for the period 1981–2010 covering Ethiopia and areas within 20–30 km (i.e., 4–6 grids) outside the boundary of Ethiopia. The data raster has a geographic coordinate system (WGS 84). The data files are stored in NetCDF format – a self-describing file format with an extension .nc that can be read and written using programming languages (Python, MATLAB, R, Ruby, IDL and Perl), programming interfaces (C, C++, Java, and Fortran), and graphical user interfaces like GIS. The dataset file is named “BCE5.nc” (stands for ‘Bias-Corrected ERA5-Land’) and has two climatic variables, the maximum and minimum daily temperature in °C. The variables of the BCE5.nc file are as follows:

• “Lat” is latitude in decimal degrees with a dimension of 260×1.
• “Lon” is longitude in decimal degrees with a dimension of 340×1.
• “Time” is a time variable in days from 1981 to 2010 with a dimension of 10957×1.
• “Tmax” is the maximum daily temperature in °C with a dimension of 260×340×10957.
• “Tmin” is the minimum daily temperature in °C with a dimension of 260×340×10957.

### 1.2 Input datasets

The input data used in the derivation of BCE5 are: the raw daily ERA5L Tx and Tn, the IOBS Tx and Tn statistics, and the Shuttle Radar Topography Mission (SRTM) digital elevation data. ERA5L [1] is a high-resolution reanalysis global dataset of several land variables governing the water and energy cycles. It is produced by re-running the land component of the European Center for Medium-Range Weather Forecast (ECMWF) ERA5 climate model on an enhanced grid resolution (0.1° × 0.1°) at hourly time steps using the atmospheric forcing from ERA5 [2] as inputs, covering the period from 1950 to 2–3 months before the present time. The period 1981–2010 was considered for the derivation of the BCE5 dataset. The IOBS data at 146 stations were obtained from the National Meteorological Service Agency (NMSA) of Ethiopia, while the data for additional 8 stations in Eritrea, Kenya, South Sudan, and Sudan were collected from the Global Historical Climatology Network (GHCN) daily dataset [3].

**Fig. 1:**
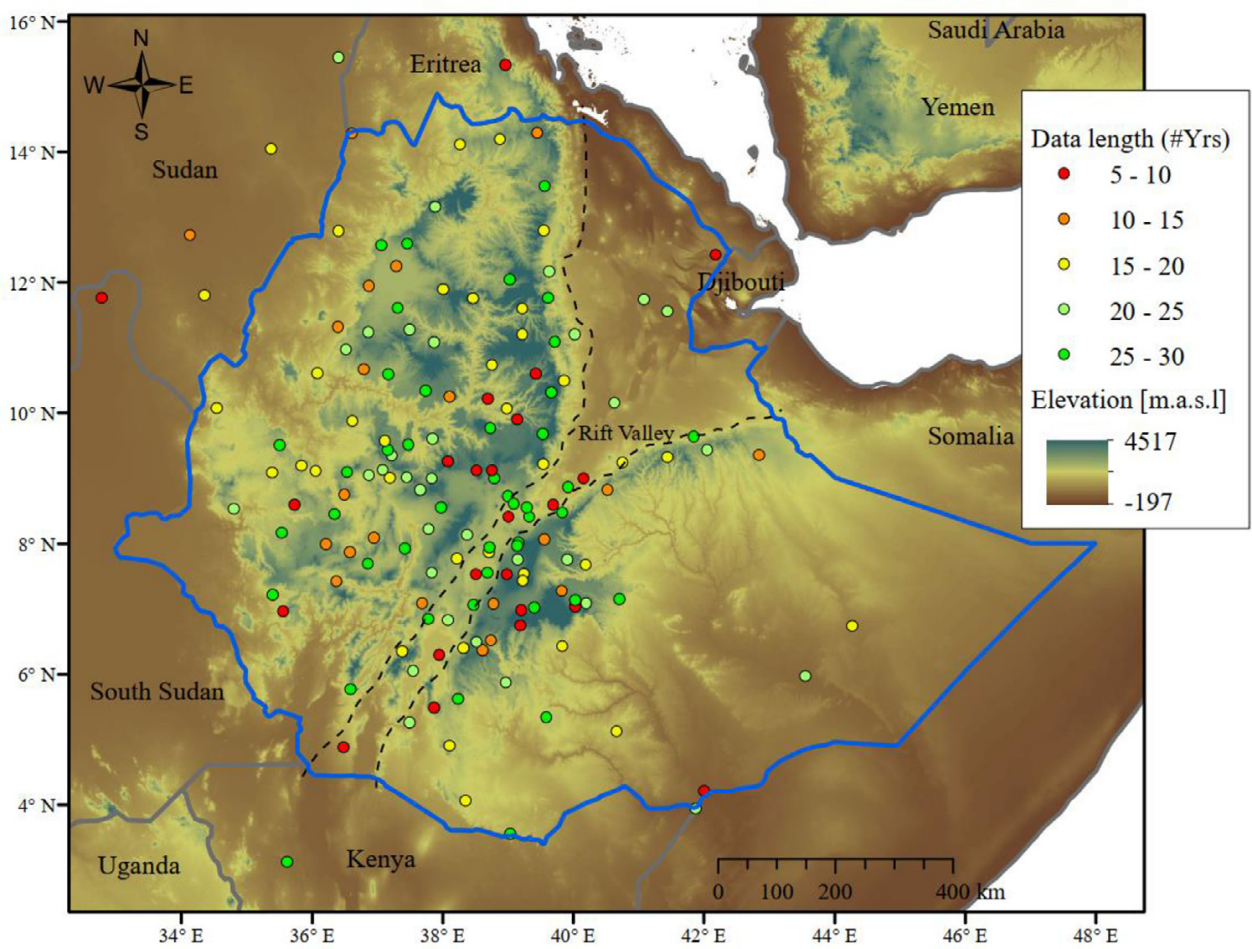
The observed temperature station network used in this study and data record lengths in years (shown by the colors of the circles) from 1981 to 2010. The background feature is the SRTM digital elevation model (https://cgiarcsi.community/ last accessed in July 2021). The dashed lines show the approximate position of the Great East African Rift Valley in Ethiopia.

**Fig. 2:**
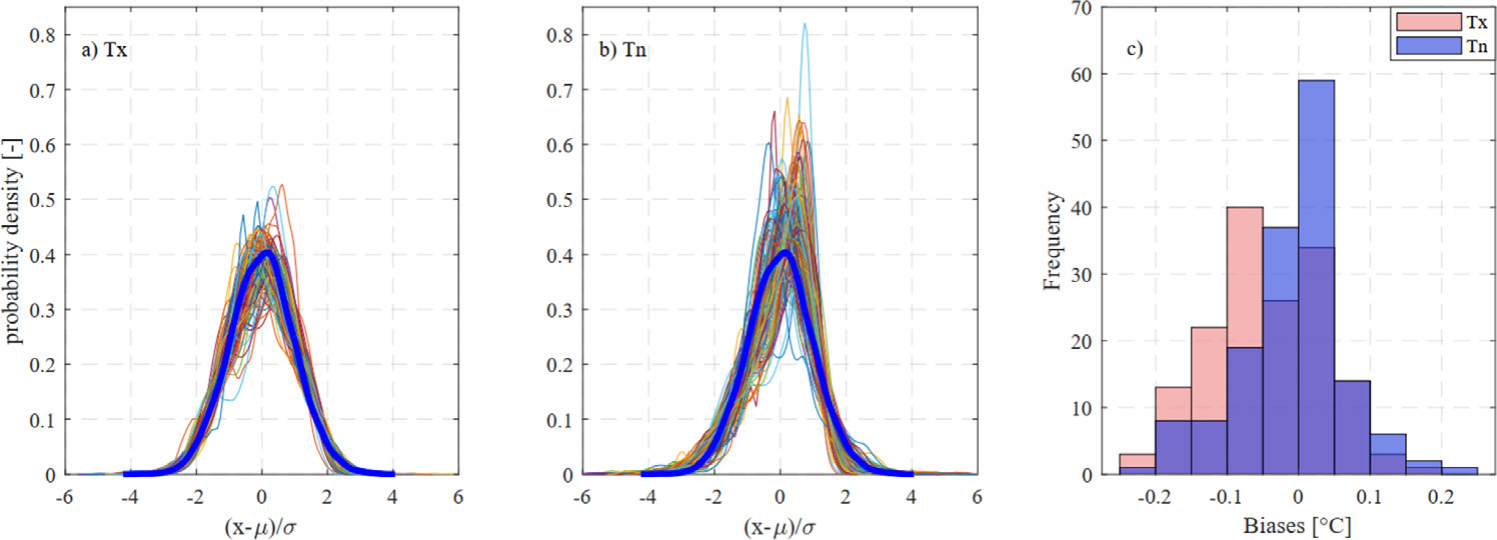
Kernel densities of standardized a) maximum daily temperature (Tx) and b) minimum daily temperature (Tn) at 146 IOBS stations over the period 1981–2010. The thick blue kernels correspond to a standardized normally distributed set of random numbers with a sample size equal to the record length of a 30-year daily temperature dataset (*n* = 10957). c) The estimated errors (sampling uncertainties) arising from the differences in record length based on the annual mean Tx and Tn.

Fig. 1 shows the digital terrain model of Ethiopia and the neighboring countries, and the spatial distribution of the IOBS stations. The IOBS datasets have enormous data gaps, which undermine the temporal continuity and overlapping of the time series. For this reason, the derivation of the BCE5 dataset was based on the first and second-order temperature statistics, rather than the complete IOBS temperature time series. The temperature data at the stations where the record is available for 5–30 years in the period 1981–2010 were used for the computation of the temperature statistics. The record length at the stations is indicated by the colors of the circles in Fig. 1. The IOBS temperature datasets follow closely a Gaussian distribution. This is illustrated in Fig. 2a and b, which indicate the probability density of the daily maximum and minimum temperatures respectively, at each station in Ethiopia. Fig. 2c reveals the estimates of uncertainties in the mean annual Tx and Tn temperature that is associated with the differences in record length.

### 1.3 Performance of the dataset

**Fig. 3:**
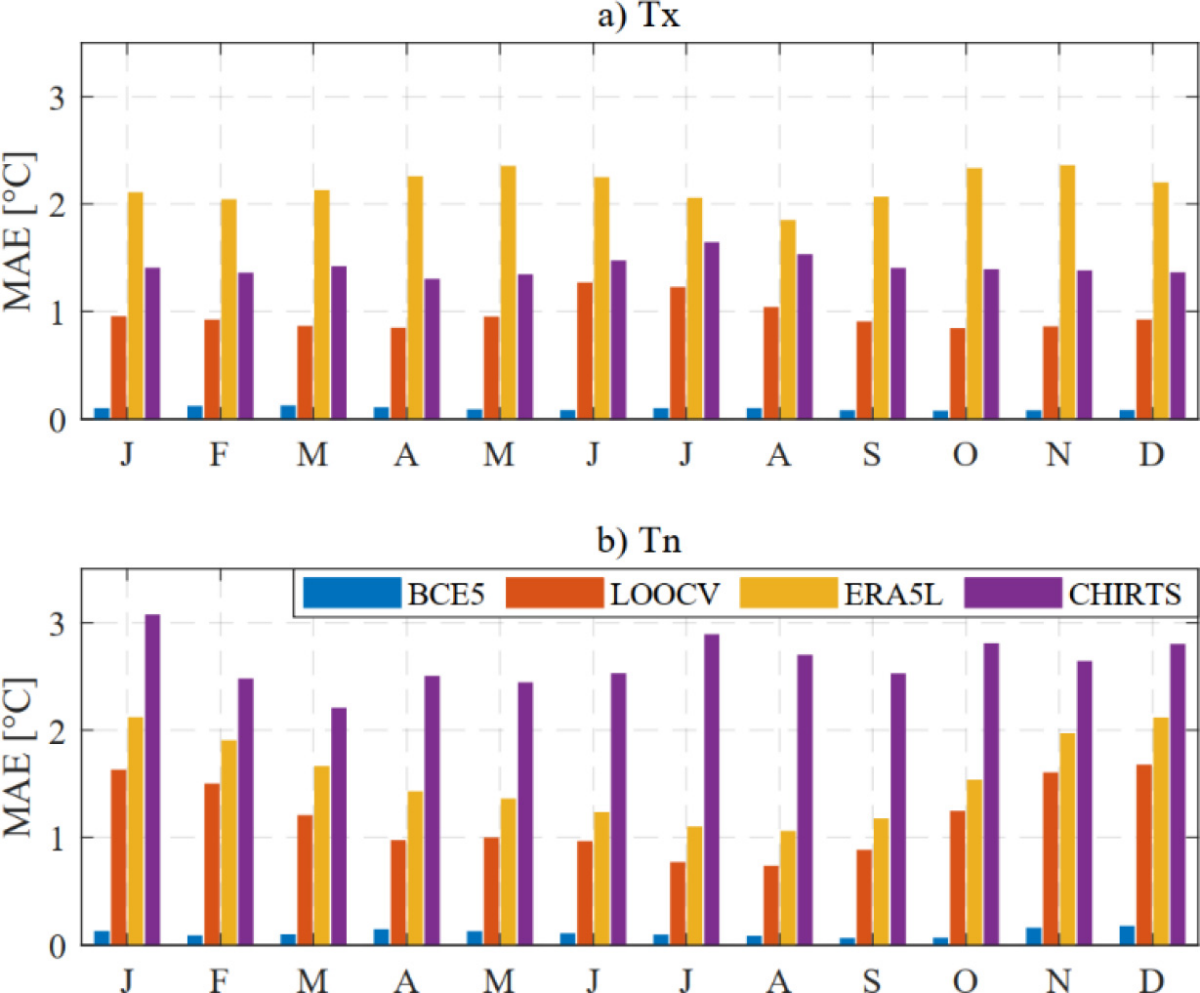
Comparison of the mean absolute errors (°C) in the monthly climatology of the BCE5 and LOOCV with ERA5L and CHIRTS for Tx (panel a) and Tn (panel b) for the period 1981–2010.

**Fig. 4:**
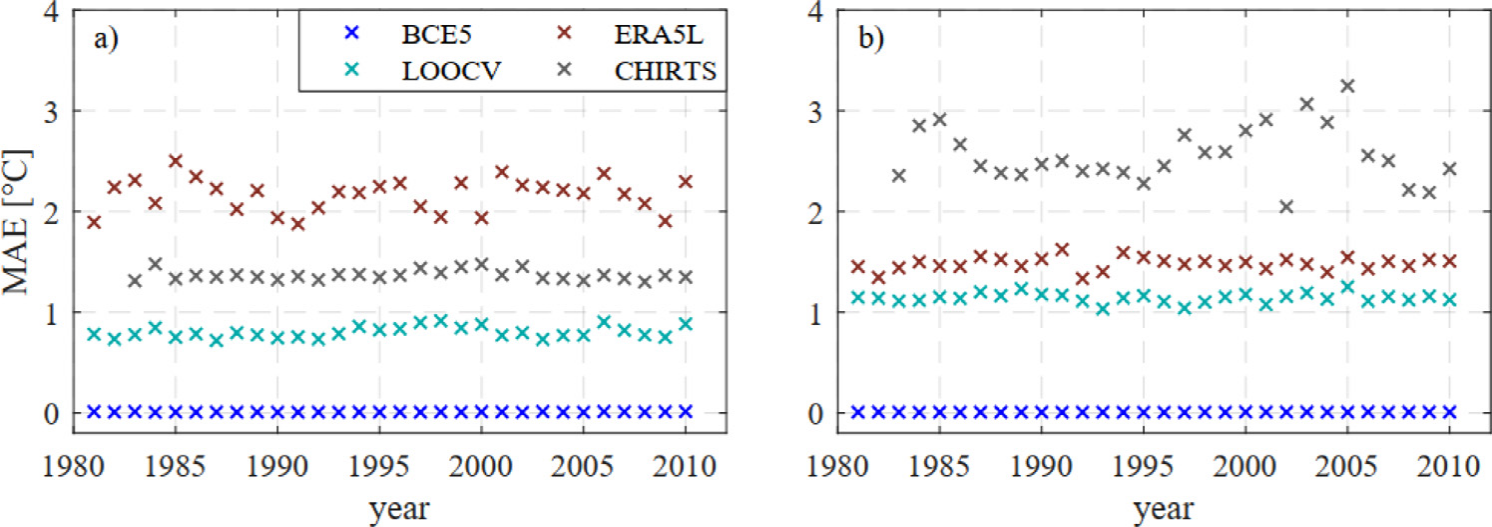
Comparison of the mean absolute errors in the annual mean BCE5 and LOOCV with ERA5L and CHIRTS for Tx (panel a) and Tn (panel b) at 146 ground stations in Ethiopia.

**Fig. 5:**
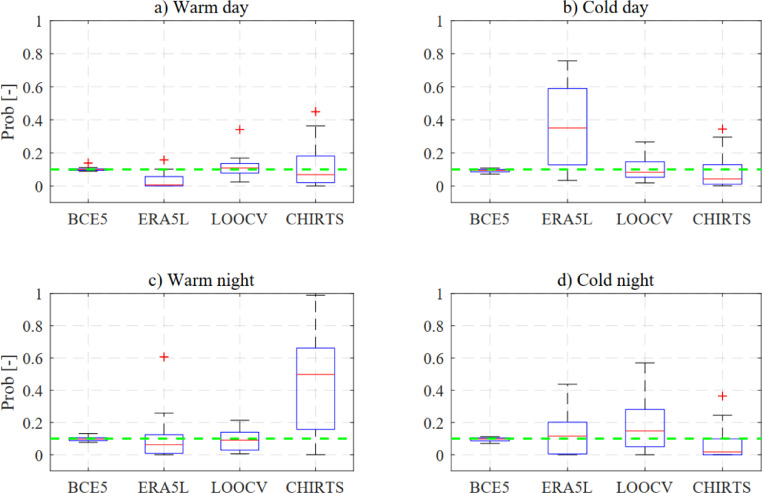
Comparison of temperature extreme probabilities in the corrected BCE5, cross-validation LOOCV, ERA5L, and CHIRTS datasets. a) warm day probability defined as Prob[Tx > 90^th^ percentile(Tx)], b) cold day probability – Prob[Tx < 10^th^ percentile(Tx)], c) probability of warm night – Prob[Tn > 90^th^ percentile(Tn)], d) probability of cold night – Prob[Tx < 10^th^ percentile(Tn)], all computed for 24 stations with continuous records of at least 25 years during 1983–2010 (chosen to match the start of CHIRTS dataset). The green dashed line shows the threshold probability of 0.1 corresponding to the frequencies computed from the IOBS data.

**Fig. 6:**
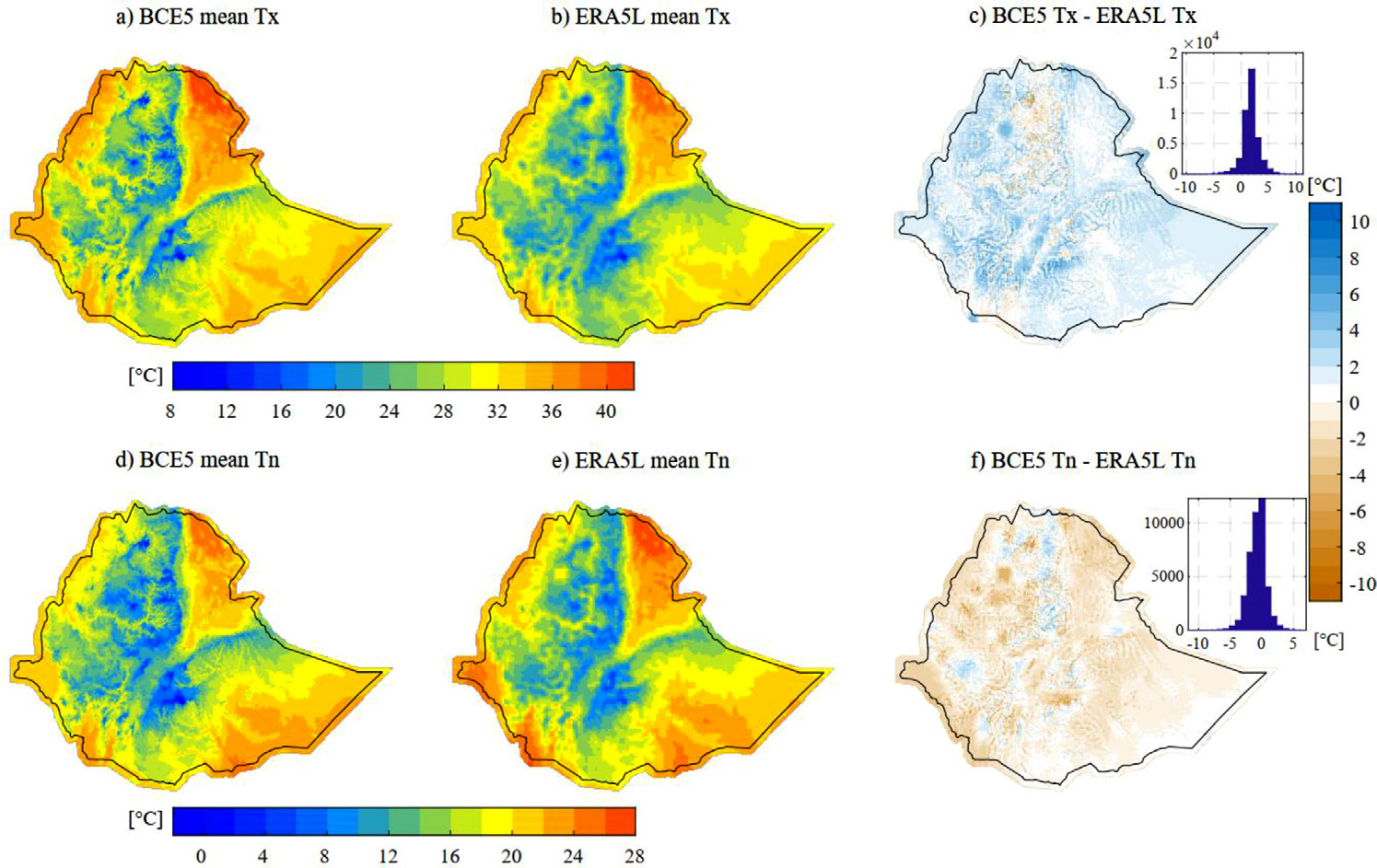
Comparison of climatological means of the bias-corrected ERA5L (BCE5) and the original ERA5L Tx (a and b) and Tn (d and e), and their differences (c and f). The insets in c and f are the histograms of the differences between BCE5 and ERA5L Tx (c) and Tn (f). The circles show the climatological means of IOBS at each of the 146 stations considered in this study.

**Fig. 7:**
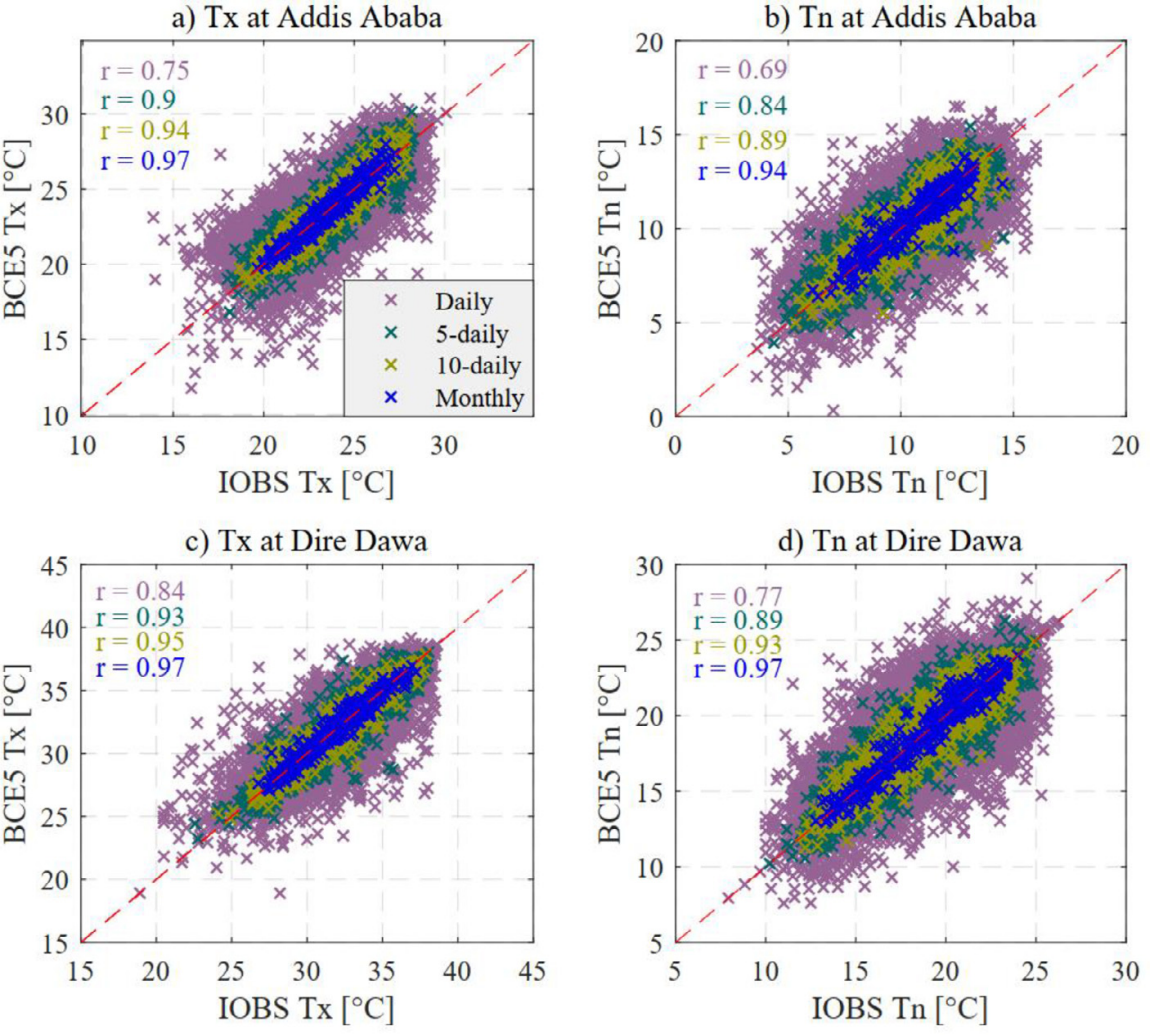
Illustrations of correlation coefficients (r) between the observed and corrected Tx and Tn datasets at different aggregation times at Addis Ababa (a and b) and Dire Dawa (c and d) stations.

The performances of the bias correction are indicated using error statistics – the mean absolute error (MAE) and root mean squared error (RMSE), temperature extreme indices and Pearson correlation coefficient. Table 1 presents the comparison of the error statistics for the daily climatologies of the derived BCE5 Tx and Tn datasets, the validation dataset that is based on the Leave-One-Out Cross Validation (LOOCV), the raw ERA5L, and the Climate Hazards group Infrared Temperature with Station (CHIRTS) – an independent gridded temperature dataset of similar temporal and spatial resolution [4]. The error statistics (MAE) for the monthly climatologies of BCE5, LOOCV, ERA5L and CHIRTS datasets are displayed in Fig. 3 whereas the MAE for the annual Tx and Tn over the period 1981–2010 is depicted in Fig. 4. Furthermore, the comparison of the extreme temperature indices, namely the probabilities of warm days (Prob[Tx > 90^th^ percentile of Tx]), warm nights (Prob[Tn > 90^th^ percentile of Tn]), cold days (Prob[Tx < 10^th^ percentile of Tx]), and cold nights (Prob[Tx < 10^th^ percentile of Tx]) [5,6] among BCE5, LOOCV, ERA5L, and CHIRTS datasets are presented in Fig. 5. The maps in Fig. 6 shows the differences between the new BCE5 and the original ERA5L datasets on the climatological time scale. In Fig. 7, the correlation between the IOBS and BCE5 temperatures at various aggregation timescales has been illustrated using the Pearson correlation coefficient at Addis Ababa and Dire Dawa stations.

**Table 1:** MAE and RMSE of the bias-corrected ERA5L (BCE5), cross-validation (LOOCV), raw ERA5L, and CHIRTS compared for daily climatology of mean and standard deviation from all IOBS locations.

	Tx				Tn			
	Mean		Std		Mean		Std	
Dataset	MAE (°C)	RMSE (°C)	MAE (°C)	RMSE (°C)	MAE (°C)	RMSE (°C)	MAE (°C)	RMSE (°C)
BCE5	0.05	0.07	0.07	0.11	0.01	0.02	0.08	0.10
LOOCV	0.68	0.91	0.27	0.37	1.07	1.27	0.48	0.63
ERA5L	2.14	2.55	0.26	0.33	1.43	1.77	0.64	0.86
CHIRTS	1.30	1.64	0.40	0.50	2.53	2.94	0.56	0.73

## 2 Experimental Design, Materials and Methods

The BCE5 dataset was derived by downscaling the ERA5L to a higher spatial resolution of 0.05°, i.e. ∼5 km, and adjusting its statistical biases using observed temperature at ground stations as a reference. The bias adjustment was made using quantile mapping (QM) where the transfer function was parameterized by the first and second-order temperature statistics summarized at the IOBS stations in Ethiopia and a few stations along the borders of the neighboring countries as discussed in Section 1.

### 2.1 Data quality control

Prior to the computation of the statistics from the IOBS, quality control measures were taken to fix the data quality issues such as outliers and homogeneity. The outliers in the datasets were checked and removed using a z-score based approach, where z-scores were computed based on the mean and standard deviation of every 30-day window, and a data point with six times or greater standard deviation from the mean over the window was considered as an outlier [7]. Inhomogeneity detections and treatments were based on manual methods [8], as automated tests were barely suitable for the detection of inhomogeneities due to the presence of significantly large and irregular data gaps in the IOBS time series. Through visual inspection of the temperature time series, three types of possible inhomogeneities were identified in Tx and Tn at some of the IOBS stations, namely: abrupt jumps in the mean, abrupt changes in the spread, and simultaneous jumps in the mean and the spread of the data. Inhomogeneities due to the abrupt jumps in the mean were corrected by shifting the arithmetic mean of the offset segment towards the mean of the time series before and after the offset segment. In the cases where the inhomogeneity involved a change in the spread of the data, the segments with the offset standard deviation were removed from the time series because the cause of these random fluctuations is difficult to determine with confidence. The removal of the segments will certainly increase the data gaps in the time series, but the effect on the statistics that are computed from the time series is minimal as illustrated in see Fig. 2c.

### 2.2 Computation of the IOBS temperature statistics

The temperature statistics (mean and standard deviation) were computed from the quality-controlled IOBS data. For the computation of the statistics and thus, the derivation of the BCE5 dataset, only the period 1981–2010, for which relatively sufficient IOBS data is available for this particular work was considered. Moreover, this period is also a standard climate period for various climate analyses, as recommended by the World Meteorological Organization (WMO) [9]. Keeping in mind the possible biases (sampling uncertainties) that could result from the differences in the record length, the climatological monthly statistics were computed from the Tx and Tn of all days of the month over the entire record length during the period 1981–2010. Similarly, the annual statistics were calculated from all days of the year in which records are available for at least 50% of the days of the year. This minimum record threshold for the annual statistics was imposed to reduce biases in the annual statistics that could arise from the temperature seasonality. This implies that for the years with no or insufficient records, the computation leaves gaps in the annual statistics. The missing values in the annual statistics were filled using a linear regression (LR) model combined with inverse generalized distance weighting interpolation [10] of four closest stations (details in Section 2.3). Note that the differences between the mean of the statistics before and after the filling result in systematic errors associated with the short records, which are also manifested in the monthly statistics. Therefore, the computed errors were added to the monthly statistics to account for some of the uncertainties that could arise from shorter record lengths.

### 2.3 Spatial interpolation of the IOBS temperature statistics

The stationary monthly and non-stationary annual temperature statistics at IOBS stations were interpolated in space onto 0.05° × 0.05° grid cells using the hybrid interpolation approach proposed by Frei [10] that combines regression and deterministic interpolation techniques in two separate steps. In the first step, the background fields of the temperature statistics were determined at every grid based on a multivariate LR model of the monthly and annual temperature statistics with elevation (*z*), and longitudinal (*x*) and latitudinal (*y*) locations as predictor variables. Accordingly, the background temperature mean $\mu_{b}$ at a target grid cell (*x,y*) was modelled as a function of z and y using $\mu_{b}\left(x,y\right)=\beta_{0}+\beta_{1}z\left(x,y\right)+\beta_{2}y\left(x,y\right)$ while the background standard deviation $\sigma_{b}$ was determined as a function of *z, y,* and *x* using $\mu_{b}\left(x,y\right)=\beta_{0}+\beta_{1}z\left(x,y\right)+\beta_{2}y\left(x,y\right)+\beta_{3}x\left(x,y\right)$, where $\beta_{0}$ is the intercept,$\;\beta_{1},\;\beta_{2},\;\beta_{3}$ are the multivariate LR model slopes, in particular $\beta_{1}$ defines the lapse rate (°C/m) whereas $\beta_{2}$ and $\beta_{3}$ are longitudinal and latitudinal location coefficients and the models were applied to determine the background Tx and Tn monthly and annual statistics. In Ethiopia, the temperature is strongly correlated with elevation and thus the predictive power of the temperature-elevation LR model is solid, making the hybrid interpolation a better approach. The coefficient of determination (R^2^) of the multivariate LR model for every month is given in Table 2.

**Table 2:** R^2^ of the linear regression model of the mean (μ) and standard deviation (σ) of Tx and Tn versus elevation (z), longitude (x), and latitude (y) based on the 154 IOBS stations.

	Maximum temperature		Minimum temperature	
Month	$\mu_{T_{x}}=f\left(y,z\right)$	$\sigma_{T_{x}}=f\left(x,y,z\right)$	$\mu_{T_{n}}=f\left(y,z\right)$	$\sigma_{T_{n}}=f\left(x,y,z\right)$
Jan	0.87	0.17	0.78	0.13
Feb	0.89	0.17	0.81	0.12
Mar	0.91	0.10	0.88	0.17
Apr	0.93	0.21	0.92	0.31
May	0.93	0.32	0.93	0.24
Jun	0.86	0.15	0.94	0.21
Jul	0.87	0.13	0.95	0.17
Aug	0.90	0.14	0.95	0.09
Sep	0.93	0.20	0.94	0.12
Oct	0.94	0.24	0.90	0.18
Nov	0.92	0.10	0.81	0.08
Dec	0.89	0.14	0.77	0.10

In the second step, the residuals of the modelled temperature statistics were computed at every station and interpolated in space using the Inverse Generalized Distance Weighting (IGDW) approach to determine the residual fields. The residual mean ($\mu_{r}$) and standard deviation ($\sigma_{r}$) at a station s are the deviations of the observed mean ($\mu_{o}$) and standard deviation ($\sigma_{o}$) from the modelled background temperature statistics and these are given as $\mu_{r}\left(s\right)=\mu_{b}\left(s\right)-\mu_{o}\left(s\right)$ for residual mean and $\sigma_{r}\left(s\right)=\sigma_{b}\left(s\right)-\sigma_{o}\left(s\right)\;$for residual standard deviation.

The IGDW interpolation approach follows the same principles of the classical Inverse Distance Weighting (IDW) interpolation but considers a non-Euclidean distance to assign weights to the stations. This is an important consideration, particularly in the context of Ethiopia to account for the effects of topographic barriers on horizontal air mass transfer between two locations in the mountainous regions. IDGW accounts for this effect to determine terrain-adjusted distance weights by penalizing the Euclidean distance between two locations L_1_ and L_2_ depending on their elevation difference. The penalized Euclidean distance is termed as a generalized distance and is computed as [10]: $D_{\lambda ,\;(L_{1}\to L_{2})}=\;\sqrt{{\left(x_{1}-x_{2}\right)}^{2}+{\left(y_{1}-y_{2}\right)}^{2}+{\left(\lambda .(z_{1}-z_{2})\right)}^{2}}$, where $D_{\lambda }$ is the generalized distance between two points, $x_{1}$ - $x_{2}$ are the longitudinal distances, $y_{1}$ - $y_{2}$ are the latitudinal distances, $z_{1}$ -$\;z_{2}$ are the elevation differences between the two locations, and λ[m/m] is a layering coefficient, i.e. a predefined free parameter that imposes an additional distance penalty per unit increase in elevation difference between the locations.

**Fig. 8:**
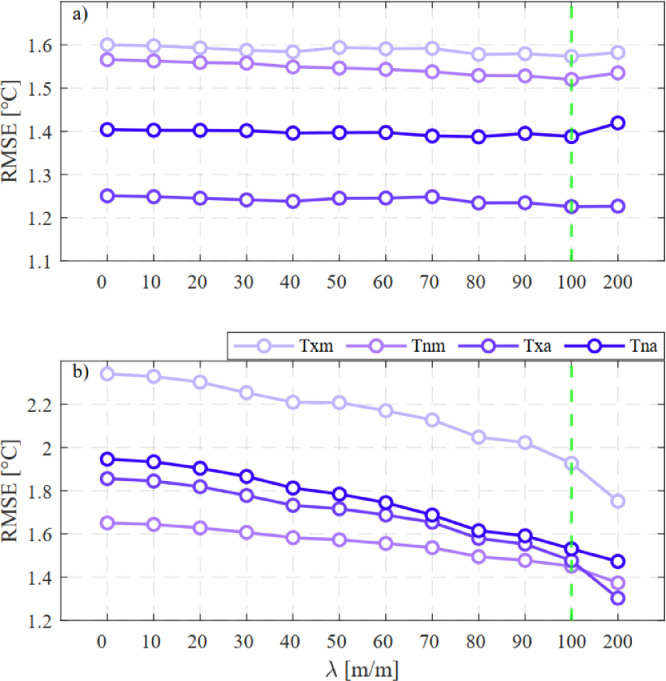
Selection of the layering coefficient λ. a) Mean cross-validation RMSE of monthly Tx and Tn (Txm and Tnm) and annual Tx and Tn (Txa and Tna) for a set of λ considering all stations. b) Mean cross-validation RMSE at highly sensitive stations, i.e., stations at which RMSE is reduced by 0.5 °C or more at λ = 100 compared to the RMSE at λ = 0. The optimum value (indicated by the green dashed line) used for the interpolation of the residual fields was chosen to be λ = 100.

The layering coefficient λ was determined based on leave-one-out cross-validation [10,11]. A set of predefined values of λ∈ [0, 10, 20, 30, 40, 50, 60, 70, 80, 90, 100, 200] were evaluated for the monthly and annual mean, and an optimal λ was chosen based on minimal root mean squared error (RMSE) as shown in Fig. 8.

Once the optimal layering coefficient was identified, the IGDW interpolation scheme was implemented to determine the residual fields at every grid: $\mu_{r}\left(x,y\right)=\frac{\sum_{i=1}^{n}\left(D_{\lambda ,i}^{-q}\mu_{r,i}\right)}{\sum_{i=1}^{n}D_{\lambda ,i}^{-q}}$ where *i* is a station and *n* is the total number of stations accounted for in the estimation of the residuals at grid (*x,y*). Four nearby stations (*n*=4) were considered optimal given the rather low station network density in the study area. An exponent of distance weight *q* = 2 was used. Note that the above equation is an IGDW scheme for the residuals of temperature mean ($\mu_{r}$). The same equation, but with λ = 0 (the classical IDW interpolation) was applied to the residuals of temperature standard deviation ($\sigma_{r}$) considering the weak correlation between elevation and the temperature variance. Finally, the gridded monthly and annual IOBS statistics that are needed to define the transfer function for the QM were obtained by adding the background and residual fields of the temperature statistics.

### 2.4 Quantile mapping

The fundamental principle of QM is to transform the distribution of a modelled variable (in our case ERA5L temperature, $T_{m}$) to the distribution of the corresponding observed variable $T_{o}$, and the concept can be formulated as $T_{o}=F_{o}^{-1}\;\left(F_{m}\left(T_{m}\right)\right)$ where $F_{o}$ is the cumulative distribution function (CDF) of $T_{o}$, which serves as a transfer function, and $F_{m}$ is the CDF of $T_{m}$ to be transformed. In our application, the CDF of $T_{o}$ is described by the interpolated statistics derived from IOBS stations as discussed in Section 2.3, and the QM bias correction is applied to $T_{m}$, which is derived from ERA5L Tx and Tn at every grid. Before the implementation of QM, the ERA5L Tx and Tn were downscaled from their original spatial resolution of 0.1° × 0.1° to the target resolution of 0.05° x 0.05° using a bilinear interpolation.

The corrections of the ERA5L Tx and Tn were performed in two steps to quantify separately the effect of de-biasing on a seasonal basis and account for nonstationarity in time. First, the temperature datasets were corrected at every grid for monthly climatology assuming stationarity, by sampling the ERA5L temperature of all days in month *m* over the entire period (1981–2010), determining the Gaussian CDF of the sub-sample (containing 847, 900, or 930 data values depending on the month), and mapping it to the CDF of the observed temperature, which is defined by the monthly statistics of the IOBS. Second, the temperature time series that was corrected for monthly climatology was additionally corrected for every year using annual sub-samples of the time series from the first step (the number of data values is 365 or 366), and the annual IOBS statistics. This step is targeted to reproduce the IOBS-based interannual variability in the bias-corrected ERA5L 2-m air temperature (hereafter BCE5).

**Fig. 9:**
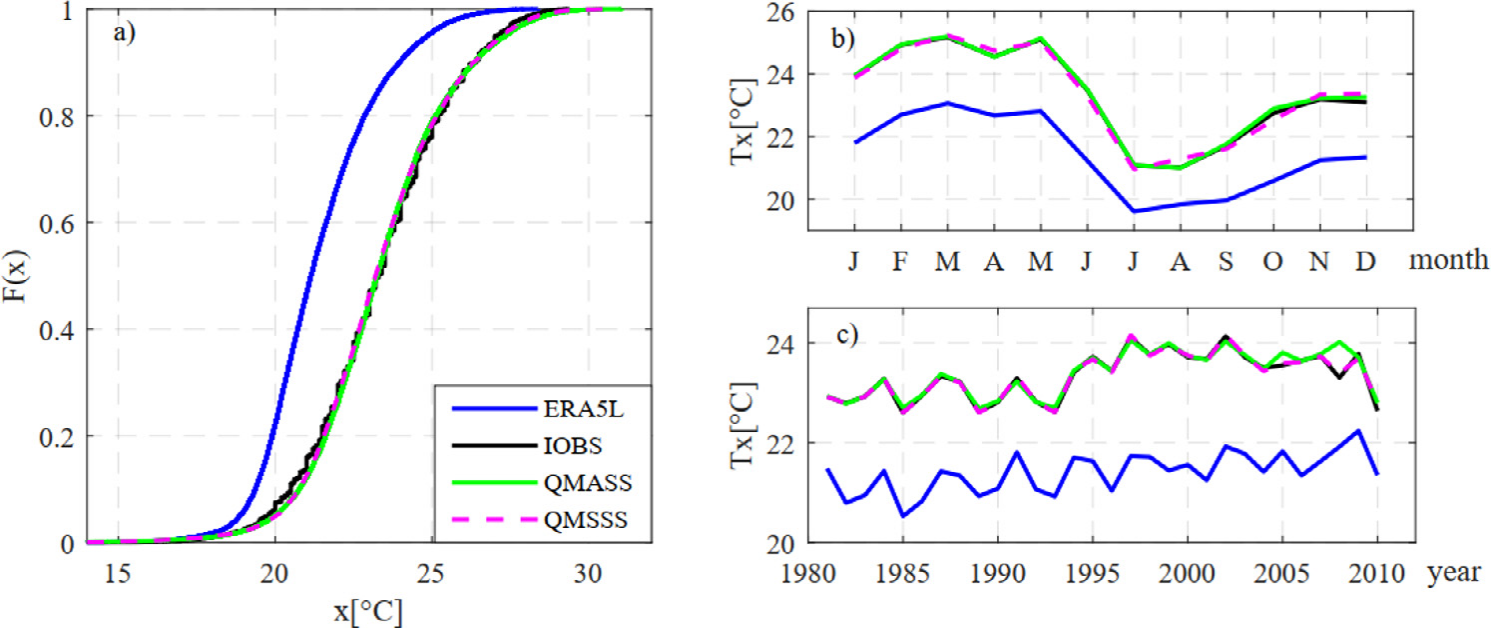
Performance comparison of sub-sampling-based QM – annual sub-samples combined with monthly climatology (QMASS, this study) and seasonal sub-samples (QMSSS, others) to IOBS and ERA5L. a) Empirical cumulative distributions, b) monthly climatology, c) interannual variability of ERA5L, IOBS, and corrected Tx, an example at Addis Ababa station for the period 1981–2010.

The QM-based corrections applied to the *annual sub-samples* combined with the corrections for monthly climatology (QMASS) in the derivation of this data yield similar results to the QM that is applied to *seasonal sub-samples* (QMSSS) by others, e.g., [12], [13], [14], [15] as demonstrated in Fig. 9. The advantage of the procedure presented here is that it allows the use of quality-controlled first and second-order statistics instead of a complete time series for the observed variables in the cases where data availability is limited. This is particularly vital for enhancing the quality of the gridded climate datasets for local applications in data-scarce regions.

### 2.5 Evaluation of the dataset

The final corrected dataset BCE5 variables Tx and Tn were evaluated using the leave-one-out cross-validation (LOOCV) approach. The evaluation was undertaken at all IOBS stations in Ethiopia which were considered in this application, using root mean squared error (RMSE) and mean absolute error (MAE) as evaluation statistics. The performance of BCE5 corresponding to the LOOCV was compared to the raw ERA5L and CHIRTS datasets for daily, monthly, annual statistics, and four extreme temperature indices – warm day, warm night, cold day and cold night probabilities, as described in Section 1.3.

In principle, QM corrects the biases in the distribution parameters, i.e. mean and standard deviation, but not the biases in the temporal sequence of the individual daily values [14]. Consequently, uncertainties are expected in the daily data. These uncertainties however sharply decrease at coarser aggregation times, for instance at pentadal (5-day) scale and coarser as illustrated in Fig. 7 Therefore, users can choose the aggregation time at which the bias-corrected dataset can be reliably used for desired applications.

## Ethics Statements

No human subjects, animal experiments, or data collected from social media platforms were involved in the derivation of the dataset presented in this paper.

## Data availability

The BCE5 daily maximum and minimum temperature dataset at 0.05° × 0.05° grid resolution for the period 1981–2010, covering Ethiopia and areas within 20–30 km (i.e., 4–6 grids) outside the boundary of Ethiopia, is freely available for scientific, commercial and non-commercial use. The dataset [16] is stored in a single NetCDF file in an open-access format and can be accessed at the ETH Zurich research collection through a permanent link (https://doi.org/10.3929/ethz-b-000546574).

## CRediT authorship contribution statement

**Mosisa Tujuba Wakjira:** Conceptualization, Data curation, Methodology, Writing – original draft. **Nadav Peleg:** Methodology, Writing – review & editing. **Paolo Burlando:** Writing – review & editing. **Peter Molnar:** Methodology, Writing – review & editing, Supervision.

## Declaration of Competing Interest

The authors declare that they have no known competing financial interests or personal relationships that could have appeared to influence the work reported in this paper.

## Data Availability

Bias-corrected and downscaled ERA5-Land 2-m air temperature dataset for Ethiopia for the period 1981-2010 (Original data) (ETH Zurich Research Collection).

## References

[1] J. Muñoz-Sabater, E. Dutra, A. Agustí-Panareda, C. Albergel, G. Arduini, G. Balsamo, S. Boussetta, M. Choulga, S. Harrigan, H. Hersbach, B. Martens, D. Miralles, M. Piles, N. Rodríguez-Fernández, E. Zsoter, C. Buontempo, J.-N. Thépaut, ERA5-Land: a state-of-the-art global reanalysis dataset for land applications, Earth Syst. Sci. Data 13. (2021) 4349–4383. doi:10.5194/essd-13-4349-2021.

[2] Hersbach H., Bell B., Berrisford P., Hirahara S., Horányi A., Muñoz-Sabater J., Nicolas J., Peubey C., Radu R., Schepers D., Simmons A., Soci C., Abdalla S., Abellan X., Balsamo G., Bechtold P., Biavati G., Bidlot J., Bonavita M., De Chiara G., Dahlgren P., Dee D., Diamantakis M., Dragani R., Flemming J., Forbes R., Fuentes M., Geer A., Haimberger L., Healy S., Hogan R.J., Hólm E., Janisková M., Keeley S., Laloyaux P., Lopez P., Lupu C., Radnoti G., de Rosnay P., Rozum I., Vamborg F., Villaume S., Thépaut J.N. (2020). The ERA5 global reanalysis. Q. J. R. Meteorol. Soc..

[3] Menne M.J., Durre I., Vose R.S., Gleason B.E., Houston T.G. (2012). An overview of the global historical climatology network-daily database. J. Atmos. Ocean. Technol..

[4] Verdin A., Funk C., Peterson P., Landsfeld M., Tuholske C., Grace K. (2020). Development and validation of the CHIRTS-daily quasi-global high-resolution daily temperature data set. Sci. Data.

[5] Donat M.G., Alexander L.V., Yang H., Durre I., Vose R., Dunn R.J.H., Willett K.M., Aguilar E., Brunet M., Caesar J., Hewitson B., Jack C., Klein Tank A.M.G., Kruger A.C., Marengo J., Peterson T.C., Renom M., Oria Rojas C., Rusticucci M., Salinger J., Elrayah A.S., Sekele S.S., Srivastava A.K., Trewin B., Villarroel C., Vincent L.A., Zhai P., Zhang X., Kitching S. (2013). Updated analyses of temperature and precipitation extreme indices since the beginning of the twentieth century: the HadEX2 dataset. J. Geophys. Res. Atmos..

[6] YIN H., SUN Y. (2018). Characteristics of extreme temperature and precipitation in China in 2017 based on ETCCDI indices. Adv. Clim. Chang. Res..

[7] Durre I., Menne M.J., Gleason B.E., Houston T.G., Vose R.S. (2010). Comprehensive automated quality assurance of daily surface observations. J. Appl. Meteorol. Climatol..

[8] (2020). https://library.wmo.int/doc_num.php?explnum_id=10352.

[9] (2017). https://library.wmo.int/doc_num.php?explnum_id=4166.

[10] Frei C. (2014). Interpolation of temperature in a mountainous region using nonlinear profiles and non-Euclidean distances. Int. J. Climatol..

[11] Hiebl J., Frei C. (2016). Daily temperature grids for Austria since 1961—concept, creation and applicability. Theor. Appl. Climatol..

[12] Maraun D. (2013). Bias correction, quantile mapping, and downscaling: revisiting the inflation issue. J. Clim..

[13] Pierce D.W., Cayan D.R., Maurer E.P., Abatzoglou J.T., Hegewisch K.C. (2015). Improved bias correction techniques for hydrological simulations of climate change. J. Hydrometeorol..

[14] Rajczak J., Kotlarski S., Schär C. (2016). Does quantile mapping of simulated precipitation correct for biases in transition probabilities and spell lengths?. J. Clim..

[15] Ruffault J., Martin-StPaul N.K., Duffet C., Goge F., Mouillot F. (2013). Projecting future drought in Mediterranean forests: bias correction of climate models matters!. Theor. Appl. Climatol..

[16] M.T. Wakjira, N. Peleg, P. Molnar, P. Burlando, Bias-corrected and downscaled ERA5-Land 2-m air temperature dataset for Ethiopia for the period 1981-2010, ETH Zurich Research Collection (May 2022). doi:10.3929/ethz-b-000546574.

